# Histologically transformed follicular lymphoma exhibits protein profiles different from both non-transformed follicular and *de novo* diffuse large B-cell lymphoma

**DOI:** 10.1038/bcj.2015.18

**Published:** 2015-03-13

**Authors:** M Ludvigsen, C Madsen, P Kamper, S J Hamilton-Dutoit, K Bendix, F d'Amore, B Honoré

**Affiliations:** 1 Department of Biomedicine, Aarhus University, Aarhus, Denmark; 2 Department of Hematology, Aarhus University Hospital, Aarhus, Denmark; 3 Institute of Pathology, Aarhus University Hospital, Aarhus, Denmark

The majority of untransformed follicular lymphoma (FL) follow an indolent clinical course and have a median overall survival that, in several series, exceeds one decade.^1, 2, 3^ Histological transformation (HT), usually to diffuse large B-cell lymphoma (DLBCL), occurs in ~30% of all patients with grade I/II FL.^4, 5, 6^ HT is usually associated with a rapidly progressive clinical course, treatment resistance and poor survival. Although HT is a well-described clinico-pathological event, the molecular mechanisms behind it are still largely unknown, particularly regarding changes in global protein expression. Moreover, no unequivocal prognostic tools have been identified to effectively predict the patients at risk of HT.

Therefore, we investigated the proteome in lymphoma samples reflecting distinct clinical settings to establish whether HT could be associated with specific protein profiles. First, we analyzed nodal FL tissue from two cohorts of patients as follows: those with a long (>10 years) indolent course and no verified transformation event (FL^nonHT^), and those characterized by the later occurrence of biopsy-verified HT (FL^HT^). Subsequently, we compared the protein expression profiles of pre- (FL^HT^) and post- (secondary DLBCL) transformation tissue samples. Finally, we compared the latter samples (secondary DLBCL) with a set of tissue biopsies from patients with DLBCL without known prior FL (*de novo* DLBCL; Figure 1a).

From each analysis, we identified differentially expressed protein spots (at least twofold and *P*<0.05): 6 spots in the comparison FL^nonHT^ vs FL^HT^ (analysis A), 9 in the comparison FL^HT^ vs secondary DLBCL (analysis B) and 28 in the comparison *de novo* vs secondary DLBCL (analysis C; Figure 1b) as listed in Table 1.

Several of the identified proteins showed complex expression patterns, for example, gelsolin, serotransferrin (TF), vimentin, hnRNP H, pyruvate kinase isozymes M1/M2 (PKM) and glyceraldehyde-3-phosphate dehydrogenase (GAPDH). Some of these were identified from more than one spot; some with approximately equal molecular masses but different isoelectric points and some from spots with lower molecular mass than expected for full-length protein. Furthermore, from some spots, various proteins were identified. The complex expression became further apparent by one-dimentional (1D) western blot (WB) analyses of these proteins.

Gelsolin showed high expression in the secondary DLBCL compared with *de novo* DLBCL (analysis C) identified from spot 4911, corresponding to the full-length protein. The gelsolin gene codes for two isoforms with similar molecular masses where only one of the isoforms was identified from the two-dimensional (2D) gels with a differential expression.^7, 8, 9^ Gelsolin expression assessed by 1D WB analysis showed a similar tendency of upregulation in the secondary DLBCL group as observed in the 2D gel analyses, although this was not significant (Supplementary Figure 1). Presumably, the expression of both the gelsolin isoforms is confined in the single major band observed in the WB and quantification with this method is thus restricted to an estimate of the total expression, as it was not possible to distinguish the two isoforms.

In the proteomic analyses, full-length TF (spot 7901) was downregulated in the secondary DLBCL group, both in comparison with FL^HT^ (analysis B) and in comparison with *de novo* DLBCL (analysis C; Figure 1b). Downregulation in secondary DLBCL was confirmed by WB analysis at borderline significance. The quantification of TF expression was, as with gelsolin, based on several isoforms in that two distinct bands were observed, which migrated close together in the WB analysis (Supplementary Figure 1). Only one spot from the 2D gels containing TF was found to be differentially expressed, which indicated that only this isoform of TF was more than twofold downregulated in secondary DLBCL. Previous studies have identified TF from multiple spots in 2D gel analyses inferring the isoformic nature of TF in lymphoma tissue.^10^ Thus, it is unknown which band from the WB analysis corresponds to the identified differential spot on the 2D gels.

Vimentin was identified from two spots, that is, one spot together with tubulin (spot 1710) that migrated corresponding to full-length vimentin/full-length tubulin with high expression in secondary DLBCL compared with *de novo* DLBCL (analysis C) and from spot 0501 with high expression in FL^nonHT^ compared with FL^HT^ (analysis A) migrating with a lower molecular mass than expected for the full-length protein (Figure 1b, Table 1). Vimentin is also known to have several isoforms that are distinguishable by 2D gel analyses.^11^ These isoforms migrate with equal molecular masses and a 1D WB method is insufficient to discriminate the distinctive isoforms, as shown in the WB analysis (Supplementary Figure 1). As two proteins were identified from the single differentially expressed spot 1710, it is not possible to determine which protein is responsible for the expression change seen. Another aspect of the putative differential expression of vimentin was found in the spot corresponding to a fragment (spot 0501). The antibody against vimentin recognized both full-length vimentin and some fragments with lower molecular masses in WB analysis (Supplementary Figure 1). The signal from the band just below the full-length protein, ~40 kDa presumably corresponding to spot 0501, was observed with a too low signal for quantification. Even longer exposure time, higher antibody concentration and higher amount of total protein were not able to generate a quantifiable signal from this band owing to the relatively higher amount of the full-length protein. The same was found for hnRNP H (spot 6202), which was identified with high expression in *de novo* DLBCL compared with secondary DLBCL (analysis C). This spot exhibited a lower molecular mass than expected for the full-length protein, and WB analysis was only able to show the fragment levels that were too low to be quantified (Figure 1b, Supplementary Figure 1). Full-length hnRNP H showed no significant differential expression in the WB analysis in agreement with no identified differentially expressed spots in the proteomic analysis with a molecular mass corresponding to full-length hnRNP H.

PKM was identified from two spots (8803 and 8805) with equal molecular masses corresponding to the full-length protein but with different isoelectric point (pI; Figure 1b). Spots 8803 and 8805 showed higher expression in secondary DLBCL in comparison with *de novo* DLBCL (analysis C) and for spot 8803 also in comparison with FL^HT^ (Figure 1b). In addition to PKM, catalase was also identified in both spots (Table 1). The expression of PKM was assessed by 1D WB analysis in which the isoforms are merged and seen as one band (Supplementary Figure 1). This combined PKM band revealed no significant difference, indicating that additional isoforms of PKM may be present. PKM was identified from spot 6408 as well. This spot migrated with a lower molecular mass than expected for full-length PKM. No apparent bands with lower molecular masses than full-length PKM were observed in the WB analysis, either because of low expression at a level below the detection limit or because the fragment was not recognized with the chosen antibody (Supplementary Figure 1).

GAPDH was identified from three spots with a complex expression, that is, spot 8301: GAPDH and fructose-biphosphate aldolase A; spot 8403: GAPDH, mitochondrial malate dehydrogenase, and annexin A2; and spot 7305: GAPDH. Low expression of spot 8301 was observed in FL^HT^ compared with FL^nonHT^ (analysis A). In the comparison of the DLBCL presentation (analysis C), spot 8403 showed high expression in secondary DLBCL and spot 7305 showed high expression in *de novo* DLBCL. Spots 7305 and 8301 migrated almost entirely together and may represent two isoforms of GAPDH with different pI and equal molecular masses, whereas spot 8403 showed a slightly higher molecular mass as well as altered pI (Figure 1b). GAPDH has previously been shown to be post-translationally modified as reviewed in Sirover^12, 13^ and Butterfield *et al.*^14^ Interestingly, in the comparisons of the two DLBCL presentations (analysis C), the GAPDH-positive spots showed high expression (spot 8403) or low (spot 7305) expression in secondary DLBCL. Thus, there were opposite expression patterns for the GAPDH isoforms in the two DLBCL presentations. The presence of several isoforms from the 2D gels was also apparent by 1D WB analysis in which the expression of GAPDH was observed as a band corresponding to the molecular mass of full-length GAPDH together with a number of lower molecular mass bands (Supplementary Figure 1).

There is a clinical need for biomarkers that may allow risk stratification of FL patients based on their risk of HT. In DLBCL, only prior evidence of previous FL can distinguish secondary transformation from *de novo* DLBCL. Therefore, a characterization of biological features that allows a distinction between these patient subsets may be useful to identify novel biomarkers of potential therapeutic relevance. In this study, we identified several protein spots showing novel differential expression with regard to possible risk of HT. The majority of differentially expressed spots were identified from a comparison of two DLBCL presentations, namely secondary and *de novo* DLBCL, implying that these morphologically similar entities differ in disease biology. We will further investigate these putative biomarkers in a larger, previously published cohort together with functional studies of the complex protein expression observed.^15^ Such supplementary studies, both in our own material and in unrelated cohorts, are warranted to establish the clinical relevance of these putative markers with regard to HT.

## Figures and Tables

**Figure 1:**
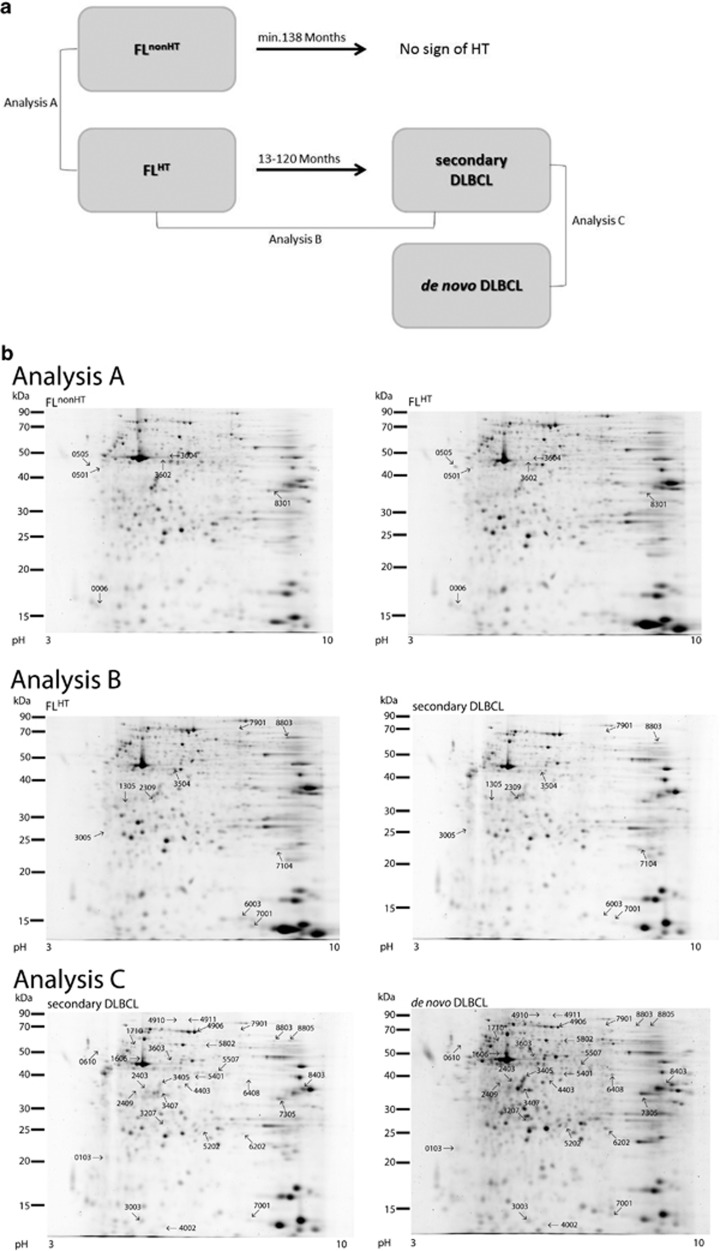
(**a**) Schematic outline of the study. Four patient groups were analyzed. FL^nonHT^: patients diagnosed with FL and followed at least 11 years with no transformation to DLBCL (*n*=5); FL^HT^: patients diagnosed with FL with subsequent transformation to DLBCL at a time point from 1 to 10 years after first diagnosis with FL (*n*=7); secondary DLBCL: patients diagnosed with DLBCL following a previously known FL diagnosis (*n*=6); and *de novo* DLBCL: patients diagnosed with DLBCL with no evidence of previous FL (*n*=9). Protein expression profiles were compared between FL^nonHT^ and FL^HT^ (analysis A), between FL^HT^ and secondary DLBCL (analysis B), and between secondary and *de novo* DLBCL (analysis C). (**b**) 2D PAGE analyses. Representative gels are shown from each group. Spots identified as differentially expressed are shown on the representative gels in the specified analyses. Analysis A: protein profiles compared between FL^nonHT^ and FL^HT^ (6 spots); analysis B: between FL^HT^ and secondary DLBCL (9 spots); and analysis C: a comparison between secondary and *de novo* DLBCL (28 spots).

**Table 1:** Identification of differentially expressed proteins

*Fold change (ratio)*	*Identified protein*	*SwissProt database code*	*Number of identified peptides*	*Mascot score*^a^	*Molecular mass (Da)*	*Spot no. on 2D gels*
*Analysis A (FL*^*HT*^*/FL*^*nonHT*^)						
0.20	Glyceraldehyde-3-phosphate dehydrogenase	G3P_HUMAN	8	612	36201	8301
	Fructose-bisphosphate aldolase A	ALDOA_HUMAN	1	74	39851	
0.25	NI					0505
0.26	Serum albumin fragment	ALBU_HUMAN	1	65	71317	3604
0.32	Vimentin fragment	VIME_HUMAN	9	480	53676	0501
0.41	ATP synthase subunit delta, mitochondrial	ATPD_HUMAN	2	133	17479	0006
	Myosin light polypeptide 6	MYL6_HUMAN	3	115	16919	
3.23	Serum albumin fragment	ALBU_HUMAN	3	147	71317	3602
						
*Analysis B (secondary DLBCL/ FL*^*HT*^)						
0.28	NI					3005
0.31	Actin^b^ fragment	ACTB_HUMAN	5	456	42052	6003
0.40	Serine/arginine-rich splicing factor 2	SRSF2_HUMAN	1	100	25461	1305^c^
0.45	HLA class I histocompatibility antigen, B-15 alpha chain	1B15_HUMAN	4	220	40648	3504
	Serpin B9	SPB9_HUMAN	9	498	43009	
	Adenosine deaminase	ADA_HUMAN	3	121	41024	
0.47	Serotransferrin	TRFE_HUMAN	1	68	79294	7901^d^
0.49	NI					7001
3.67	Serine/arginine-rich splicing factor 1	SRSF1_HUMAN	2	74	27842	2309
2.37	Peroxiredoxin-1	PRDX1_HUMAN	2	79	22324	7104
2.21	Pyruvate kinase isozymes M1/M2	KPYM_HUMAN	21	1465	58470	8803d,^e^
	Catalase	CATA_HUMAN	2	126	59947	
						
*Analysis C (secondary DLBCL/de novo DLBCL)*						
0.26	Macrophage-capping protein fragment	CAPG_HUMAN	1	65	38760	3207^d^
0.26	Coronin-1A	COR1A_HUMAN	6	388	51678	5802
0.31	Tubulin^b^ fragment	TBA1_HUMAN	4	306	50820	5401
	Alpha-enolase fragment	ENOA_HUMAN	2	97	47481	
0.33	Actin^b^	ACTA_HUMAN	1	77	42367	2409^c^
0.33	Tubulin fragment	TBA1B_HUMAN	3	239	50804	2403^e^
0.36	Cofilin-1 fragment	COF1_HUMAN	1	75	18719	3003
0.37	Serum albumin fragment	ALBU_HUMAN	2	93	71317	5202
0.42	Serum albumin fragment	ALBU_HUMAN	5	316	71317	3603^e^
0.45	Heterogeneous nuclear ribonucleoprotein H fragment	HNRH1_HUMAN	4	276	49484	6202
0.45	HLA class I histocompatibility antigen^b^	1A02_HUMAN	3	114	41181	5507
	HLA class I histocompatibility antigen^b^	1B15_HUMAN	2	112	40648	
0.45	Pyruvate kinase isozymes M1/M2 fragment	KPYM_HUMAN	6	251	58522	6408
0.45	Serotransferrin	TRFE_HUMAN	1	68	79294	7901^d^
0.46	NI					7001^d^
0.46	NI					4002
0.46	Glyceraldehyde-3-phosphate dehydrogenase	G3P_HUMAN	4	292	36201	7305
0.47	Tubulin^b^ fragment	TBB5_HUMAN	6	324	50095	4403
0.48	Actin^b^	ACTB_HUMAN	6	523	42052	3405
	Pyruvate dehydrogenase E1 component subunit beta, mitochondrial	ODPB_HUMAN	2	140	39550	
3.45	NI					4910
3.33	Gelsolin	GELS_HUMAN	2	72	86043	4911
3.13	Brain acid soluble protein 1	BASP1_HUMAN	4	163	22680	0610
2.70	Glyceraldehyde-3-phosphate dehydrogenase	G3P_HUMAN	7	584	36201	8403
	Glyceraldehyde-3-phosphate dehydrogenase	G3P_NEUCR	1	52	36384	
	Malate dehydrogenase, mitochondrial	MDHM_HUMAN	7	370	35937	
	Annexin A2	ANXA2_HUMAN	1	56	38873	
2.50	Guanine nucleotide-binding protein G(I)/G(S)/G(T) subunit beta	GBB1_HUMAN	3	182	38151	3407^e^
2.44	Pyruvate kinase isozymes M1/M2	KPYM_HUMAN	20	1374	58470	8805^e^
	Catalase	CATA_HUMAN	1	48	59947	
2.38	Vimentin	VIME_HUMAN	9	446	53676	1710
	Tubulin^b^	TBA1B_HUMAN	4	204	50804	
2.33	Serum albumin	ALBU_HUMAN	16	1030	71317	4906
2.33	Pyruvate kinase isozymes M1/M2	KPYM_HUMAN	21	1465	58470	8803d,^e^
	Catalase	CATA_HUMAN	2	126	59947	
2.17	Perilipin-3	PLIN3_HUMAN	2	240	47046	1606
2.04	Proteasome subunit beta type-9	PSB9_HUMAN	4	260	23364	0103

Abrreviations: DLBC, diffuse large B-cell lymphoma; 2D, two-dimensional; FL, follicular lymphoma; HT, histological transformation; no., number; NI, Not identified.

a Mascot score: Ion score is −10*Log(P), where P is the probability that the observed match is a random event.

b A number of variants was found. Top hit listed.

c Contaminated with tubulin from previous samples.

d Identified from analysis B as well as analysis C.

e Few additional significant peptides were found with scores just above significance level.
