# Supplementary material for: Histologically transformed follicular lymphoma exhibits protein profiles different from both non-transformed follicular and de novo diffuse large B-cell lymphoma
Source: Blood Cancer J. 2015 Mar 13;5(3):e293–. doi: 10.1038/bcj.2015.18 (PMC4382659; doi:10.1038/bcj.2015.18)
Supplement: Supplementary Information [file bcj201518x1.docx]

Supplementary information

Histologically transformed follicular lymphoma exhibits protein profiles different from both non-transformed follicular and *de novo* diffuse large B-cell lymphoma

Ludvigsen, M *et al*

**PATIENT AND METHODS**

The present study was approved by the Regional Research Ethics Committee and by the Danish Data Protection Agency.

**Patients**

A cohort of 27 patients diagnosed at the Department of Hematology, Aarhus University Hospital, Denmark between 1994 and 2007 was identified (FL^nonHT^: 1996-1999, FL^HT^: 1994-2007, secondary DLBCL: 1998-2003, 'de novo' DLBCL: 1997 – 2007). Five patients were diagnosed with FL and followed at least 11 years without HT (FL^nonHT^). Seven patients were diagnosed with FL and subsequent HT within 1 to 10 years from primary diagnosis (FL^HT^). No patients in the FL^nonHT^ group were treated with Rituximab. One patient in the FL^HT^ received R-CVP at FL stage (F414). Six patients were diagnosed with DLBCL with a previously known FL diagnosis (secondary DLBCL) and nine patients were diagnosed with DLBCL with no knowledge of previous lymphoid malignancy (*de novo* DLBCL). In two FL^HT^ cases (patient F43 and F451), sequential samples from the FL and DLBCL stage, respectively, were available. Clinico-pathological parameters (Table 1, supplementary) as well as treatment and outcome data were obtained from the Danish lymphoma registry LYFO (lymphoma.dk) and from patient records.

**Tissue samples**

Frozen tissue samples were handled as previously described.[^1^](#_ENREF_1)^,^ [^2^](#_ENREF_2) For all three groups (FL^nonHT^, FL^HT^ and *de novo* DLBCL), pretreatment tissue samples were used. Frozen tissue samples from secondary DLBCL derived from biopsies taken at the time of transformation and prior to start of antineoplastic therapy.

Hematoxylin-eosin and immunohistochemically stained tissue sections from formalin-fixed paraffin-embedded tumor samples were submitted for diagnostic review to experienced hematopathologists (SHD, KB).

**Two-dimensional polyacrylamide gel electrophoresis (2D-PAGE)**

Tissue pre-treatment and 2D-PAGE procedures together with staining were performed as previously described.[^1^](#_ENREF_1)^,^ [^2^](#_ENREF_2)

**Data analysis**

Proteomic analyses were performed by comparing patient sub-cohorts as shown in Figure 1A in the text. Gel images were imported into the PDQuest Advanced 2D Gel Analysis Software Version 8.0 (Bio-Rad, Berkeley, CA, USA) and analyzed as previously described.[^1^](#_ENREF_1)^,^ [^2^](#_ENREF_2) All matches were critically evaluated and the necessary editions and corrections were performed manually. Spots with significant (Mann-Whitney U test, P < 0.05), but at least 2-fold, differential expression were selected.

**Protein identification by liquid chromatography tandem mass spectrometry (LC-MS/MS)**

Sample preparation and MS analysis were essentially performed as previously described.[^1^](#_ENREF_1)^,^ [^2^](#_ENREF_2)^,^ [^3^](#_ENREF_3) However, the high performance liquid chromatography procedure for peptide separation was slightly modified. The dried fragmented protein samples were dissolved and peptide separation before MS/MS analysis was performed using a nanoliquid chromatography system, EASY-nLC II (Thermo Scientific, Waltham, MA, USA). The samples were then analyzed on a Q-TOF Premier mass spectrometer (Waters, Milford, MA, USA). The processed data were analyzed by searching the Swiss-Prot Database (version 2013_05 or 2013_10 containing 540,052 or 541,561 sequences, respectively) using the online version of the Mascot MS/MS Ion Search facility (Matrix Science, Ltd., <http://www.matrixscience.com>).[^4^](#_ENREF_4) For database searches, doubly, triply, and quadruply charged ions with up to 2 missed cleavages, one fixed modification, carbamidomethyl (Cys), variable modifications, oxidation (Met, His, Trp), dioxidation (Met) or phosphorylation (Ser, Thr, Tyr), a peptide mass tolerance of 20 ppm, and an MS/MS tolerance of 0.05 Da were allowed. Background signals from environmental peptides, such as keratins, cingulin, fillaggrin-2, dermicidin, desmoglein-1, trypsin, BSA and casein were disregarded. If only one significant peptide was found, the spectrum was critically evaluated and discarded if the quality was found insufficient. If proteins were identified based on two non-significant peptides, these were also discarded. Generally these identifications were sensitive to changes in search criteria (modifications). All robustly identified proteins are reported in Table 1 in the text.

**1D Western blot (WB) analysis**

Where possible, WB was used for immunological visualization of differentially expressed proteins. Tissue preparation was performed as previously described. Ten µg of each sample was loaded on the gels. Tumor sample with adequate amount of protein was not available for the F43 specimen. The WB procedure and subsequent quantification were performed as previously reported.[^1^](#_ENREF_1) As described previously, “housekeeping” proteins are often used as control because the expression of these proteins is assumed to be at a constant level under varying conditions. However, there is increasing evidence from proteomic studies that “housekeeping” proteins such as actin, tubulin, and GAPDH may be inadequate as internal standards as their expression can be affected by a large number of factors.[^3^](#_ENREF_3)^,^ [^5^](#_ENREF_5)^,^ [^6^](#_ENREF_6)^,^ [^7^](#_ENREF_7) In the present study and as well as our previous studies,[^1^](#_ENREF_1)^,^ [^2^](#_ENREF_2) we have identified several differentially expressed protein spots containing peptides from actin, tubulin and GAPDH. Therefore, “housekeeping” proteins were not considered adequate as reference proteins in the present study. Instead, we normalized the WBs to the total protein concentration present in the tissue sample.[^1^](#_ENREF_1)^,^ [^2^](#_ENREF_2)^,^ [^8^](#_ENREF_8)

Primary antibodies were purchased from commercial suppliers: anti-vimentin (sc-7558-R, Santa Cruz Biotechonology, Dallas,Texas, USA), anti-GAPDH (HPA040067, Atlas Antibodies, Stockholm, Sweden), anti-serotransferrin (anti-transferrin ab1223, diluted 1:30, Abcam, Cambridge, UK), anti-PKYM (HPA029501, Atlas Antibodies, Stockholm, Sweden), anti-gelsolin (ABS 017-20, Waltham, MA, USA), or previously made in our lab: anti-hnRNP H.[^9^](#_ENREF_9) Secondary antibodies were HRP-conjugated secondary antibodies (P217 and P260, DAKO, Glostrup, Denmark)).

**TABLES**

Table 1, supplementary - Selected clinico-pathological features

| **Pt ID** | **Months**  **to HT** | **Age, years**  **(at primary diagnosis / at transformation)** | **Follow-up from time of primary diagnosis, month** | **FL histological grade (WHO)** | **Ann Arbor stage** | **FLIPI** | **IPI** |
| --- | --- | --- | --- | --- | --- | --- | --- |
| **FL^nonHT^** |  |  |  |  |  |  |  |
| FL245 | - | 62 | 147 | 2 | 3 | 3 | - |
| FL393 | - | 56 | 138 | 1 | 3 | 3 | - |
| FL335 | - | 49 | 151 | 1 | 4 | 4 | - |
| FL199 | - | 45 | 154 | 1 | 3 | 3 | - |
| FL521 | - | 54 | 177 | 2 | 1 | 1 | - |
| **FL^HT^** |  |  |  |  |  |  |  |
| FL184 | 13 | 57/58 | 15 | 1 | 4 | 4 | - |
| FL257 | 112 | 66/75 | 123 | 2 | 4 | 4 | - |
| FL447 | 90 | 25/33 | 124 | 2 | 4 | 3 | - |
| FL271 | 95 | 59/66 | 173 | 1 | 2 | 2 | - |
| FL414 | 21 | 65/66 | 35 | 2 | 4 | 3 | - |
| FL43 | 34 | 48/50 | 138 | 2 | 4 | 2 | - |
| FL451 | 14 | 53/54 | 152 | 1 | 4 | 3 | - |
| **Secondary DLBCL** | |  |  |  |  |  |  |
| FL43-D | 34 | 48/50 | 104 | - | 3 | 2 | - |
| FL451-D | 14 | 53/54 | 138 | - | 4 | 3 | - |
| FL275 | 54 | 43/48 | 15 | - | 4 | 4 | - |
| FL39 | 16 | 55/59 | 176 | - | 4 | 3 | - |
| FL91 | 120 | 48/58 | 123 | - | 4 | 4 | - |
| FL72 | 16 | 54/55 | 173 | - | 4 | 2 | - |
| ***De novo* DLBCL** | |  |  |  |  |  |  |
| D907 | - | 82 | 48 | - | 1 | - | 3 |
| D174 | - | 79 | 78 | - | 3 | - | 3 |
| D333 | - | 59 | 107 | - | 4 | - | 3 |
| D478 | - | 53 | 147 | - | 1 | - | 1 |
| D709 | - | 59 | 27 | - | 4 | - | 2 |
| D971 | - | 80 | 78 | - | 4 | - | 3 |
| D1038 | - | 54 | 142 | - | 4 | - | 3 |
| D204 | - | 66 | 166 | - | 1 | - | 2 |
| D810 | - | 49 | 13 | - | 4 | - | 3 |

**FIGURES**

Figure 1, supplemetary

**
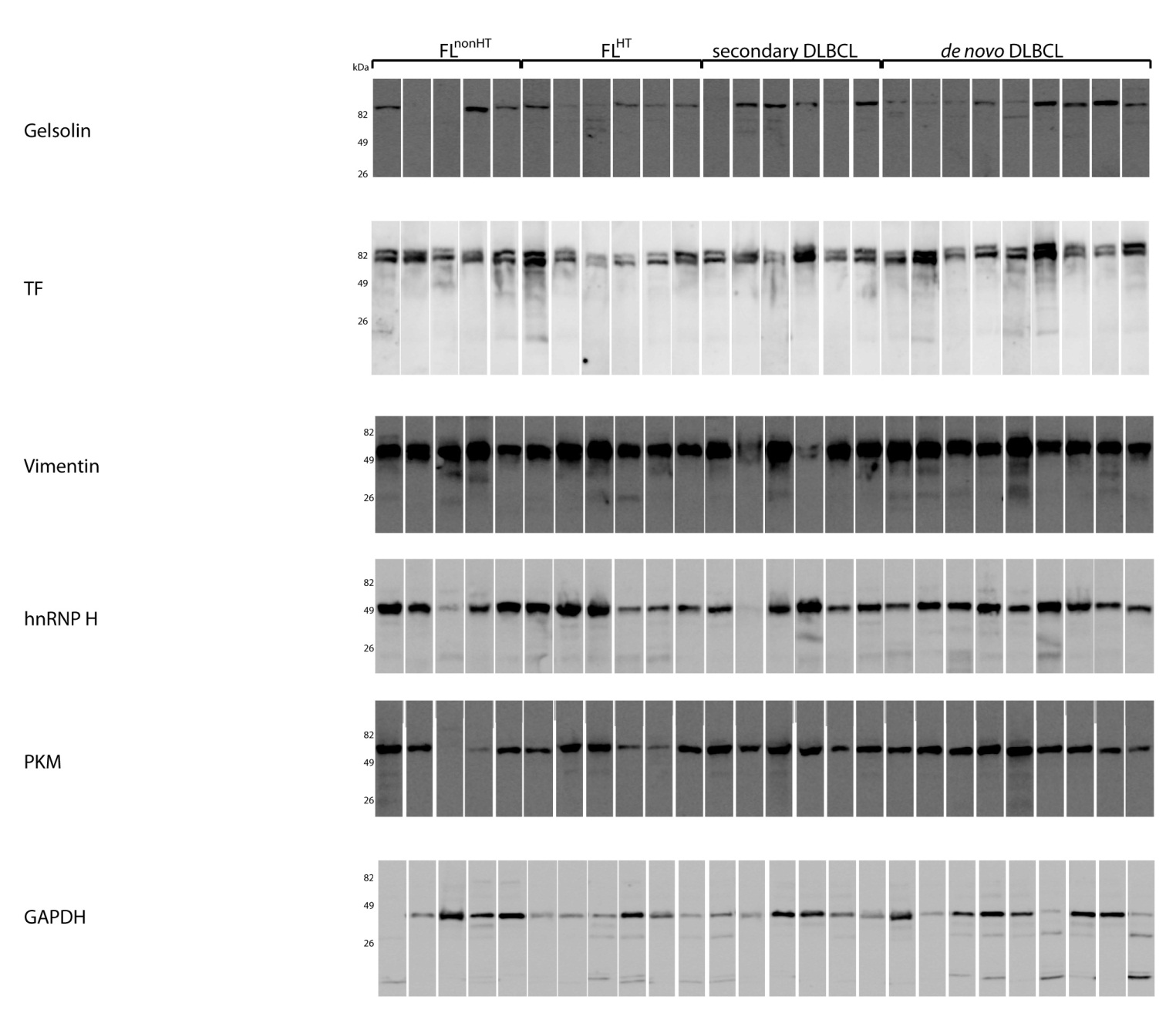
**

Figure 1, supplementary. Protein expression by 1D-WB. WBs of selected identified differentially expressed proteins from the proteomic analyses: gelsolin, TF, vimentin, hnRNP H, PKM, and. Due to the number of patients in the study, the WB analysis was done on 4 separate WB. Patient sample F245 (FL^nonHT^) was used as reference and was loaded on each gel. Moreover, each blot contained samples from the four subsets. Following quantification, pixel intensities from the specified bands were normalized against the pixel intensities of F245 in each WB. FL^nonHT^, patients diagnosed with FL with no transformation to DLBCL; FL^HT^, patients diagnosed with FL and experienced transformation to DLBCL; secondary DLBCL, patients diagnosed with DLBCL with a previously known FL diagnosis; *de novo* DLBCL, patients diagnosed with DLBCL with no knowledge of previous FL.

**REFERENCES**

1. Kamper P, Ludvigsen M, Bendix K, Hamilton-Dutoit S, Rabinovich GA, Moller MB*, et al.* Proteomic analysis identifies galectin-1 as a predictive biomarker for relapsed/refractory disease in classical Hodgkin lymphoma. *Blood* 2011, **117**(24)**:** 6638-6649.

2. Ludvigsen M, Kamper P, Hamilton-Dutroit SJ, Bendix K, Moller MB, d'Amore FA*, et al.* Relationship of intratumoural protein expression patterns to age and Epstein-Barr virus status in classical Hodgkin lymphoma. *Eur J Haematol* 2014; e-pub ahead of print 11 October 2014; doi: 10.1111/ejh.12463

3. Honore B, Buus S, Claesson MH. Identification of differentially expressed proteins in spontaneous thymic lymphomas from knockout mice with deletion of p53. *Proteome science* 2008, **6:** 18.

4. Perkins DN, Pappin DJ, Creasy DM, Cottrell JS. Probability-based protein identification by searching sequence databases using mass spectrometry data. *Electrophoresis* 1999, **20**(18)**:** 3551-3567.

5. Ferguson RE, Carroll HP, Harris A, Maher ER, Selby PJ, Banks RE. Housekeeping proteins: a preliminary study illustrating some limitations as useful references in protein expression studies. *Proteomics* 2005, **5**(2)**:** 566-571.

6. Colell A, Green DR, Ricci JE. Novel roles for GAPDH in cell death and carcinogenesis. *Cell Death Differ* 2009, **16**(12)**:** 1573-1581.

7. Dumontet C, Jordan MA. Microtubule-binding agents: a dynamic field of cancer therapeutics. *Nat Rev Drug Discov* 2010, **9**(10)**:** 790-803.

8. Ludvigsen M, Kamper P, Sorensen BS, Moller MB, Bendix K, Hamilton-Dutoit SJ*, et al.* Differential protein expression of peroxiredoxin-1 in classical Hodgkin Lymphoma: a possible correlation to clinical behaviour. *Hematol Oncol* 2014.

9. Honore B, Vorum H, Baandrup U. hnRNPs H, H' and F behave differently with respect to posttranslational cleavage and subcellular localization. *FEBS Lett* 1999, **456**(2)**:** 274-280.
